# Muscle fibre type shift in COPD: Adaptive, maladaptive or a bit of both?

**DOI:** 10.1113/EP092126

**Published:** 2025-03-03

**Authors:** Jacob Peter Hartmann, Hannah G. Caldwell, Ulrik Winning Iepsen

**Affiliations:** ^1^ Centre for Physical Activity Research Copenhagen University Hospital – Rigshospitalet Copenhagen Denmark; ^2^ Department of Clinical Physiology and Nuclear Medicine Copenhagen University Hospital – Rigshospitalet Copenhagen Denmark; ^3^ Department of Nutrition, Exercise and Sports, The August Krogh Section for Human Physiology University of Copenhagen Copenhagen Denmark; ^4^ Department of Anesthesia and Intensive Care Copenhagen University Hospital – Hvidovre Hospital Hvidovre Denmark

Aristotle as well as numerous other philosophers have pondered over the chicken‐and‐egg paradox, asking which came first. For chronic obstructive pulmonary disease (COPD) patients, this dilemma plays out in the body: does the disease's progression lead to reduced physical activity, causing muscle dysfunction, a major systemic consequence of COPD contributing to exercise limitations, or does the weakening and dysfunction of the muscles come first, spiralling patients into inactivity?

Across studies, a shift in quadriceps muscle fibre type (from oxidative type I fibres to glycolytic type II fibres) has been linked to the severity of COPD (Figure 1) (Gosker et al., 2002). This fibre‐type transition likely causes exercise‐induced muscle fatigue (Amann et al., 2010), which may be driven by impaired oxygen transport to the working muscle. Interestingly, skeletal muscle adaptations are not consistent across the body in COPD. The respiratory muscles adapt in the opposite direction by fibre‐type transformation towards a higher proportion of type I fibres in the diaphragm, which is also associated with COPD severity (Figure 1), whereas the muscles of the upper extremity remain unaffected (Gea et al., 2001; Hernández et al., 2003; Levine et al., 2006, 2003), and hence, chronic tissue hypoxia may not be the main stimulus.

**FIGURE 1:**
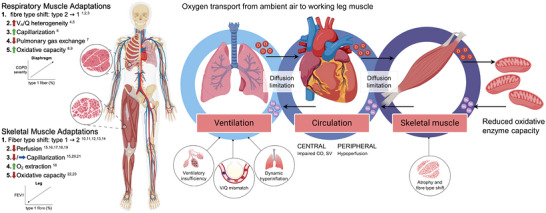
Comparing and contrasting skeletal and respiratory muscle adaptations in COPD. The respiratory muscles adapt by fibre‐type shift towards a higher proportion of type I fibres in the diaphragm; this is associated with COPD severity and likely impacted by increased alveolar ventilation–perfusion heterogeneity and impaired pulmonary gas exchange. In contrast, several studies report an opposite shift in quadriceps muscle fibre type (from oxidative type I fibres to glycolytic type II fibres), which is related to the severity of COPD. The implications of this fibre‐type shift likely include exercise‐induced muscle fatigue, which may be driven by impaired oxygen transport to working muscle. CO, cardiac output; COPD, chronic obstructive pulmonary disease; SV, stroke volume; VA/Q, alveolar ventilation/perfusion. This figure was created with BioRender.com

The evidence mentioned above typically comes from cross‐sectional studies of skeletal muscle (Figure 1), which have limited spatial resolution or information on metabolic function. To better understand fibre‐type distribution and muscle function, enzymes involved in muscle metabolism have been investigated. Some reports show that the quadriceps exhibit a less oxidative and more glycolytic profile in COPD (Allaire et al., 2004; Maltais et al., 2014, 2000), while the diaphragm relies more on oxidative phosphorylation than glycolytic metabolism (Ribera et al., 2012).

Another important component of COPD‐related muscle adaptations is the capillary network of the skeletal muscle fibres, an attribute important for adequate muscle function (Poole et al., 2022). Studies investigating skeletal muscle capillarization report a decreased capillary‐to‐fibre ratio (Gouzi et al., 2013) or alterations in the muscle‐to‐capillary interface (Eliason et al., 2010), in contrast to others which found no difference in capillarization between COPD and healthy (Iepsen et al., 2017; Richardson et al., 2004). The divergent results may be attributed to differences in techniques used across studies and the lack of integrative information at the whole‐muscle level (Figure 1), making it challenging to draw firm conclusions about fibre‐type shifting and the associated metabolic function.

## 1 MUSCLE OXIDATIVE CAPACITY – THE HIGHER, THE BETTER?

Patients with COPD exhibit a lower exercising leg blood flow across disease severities and some studies also report an attenuated muscle oxygen diffusing capacity (Broxterman et al., 2020, 2021; Hartmann et al., 2022; Iepsen et al., 2017; Mohammad et al., 2026). The reason for the decreased leg muscle oxygen conductance in COPD remains to be determined, but perhaps it relates to the local changes in the muscle or the more central components in the oxygen transport cascade (Figure 1). In the diaphragm, on the other hand, mitochondrial capacity may be upregulated in COPD (Ribera et al., 2012), perhaps as an adaptive response to the chronically elevated respiratory workload in these patients. In theory, to preserve O_2_ delivery to match the high demand of the respiratory muscle during exercise, perfusion of the locomotor muscles may be restricted (Sheel et al., 2001; St Croix et al., 2000). This idea is supported by studies in patients with COPD showing that unloading of the respiratory muscle work during exercise increases working leg muscle oxygenation (Borghi‐Silva et al., 2008; Dempsey, 2019). Again, this apparent redistribution of blood volume during exercise towards respiratory muscle is not a universal finding in COPD as another study showed that there was a decrease in perfusion of the intercostal muscles during heavy exercise (Vogiatzis et al., 2020). Importantly, it is the diaphragm, not intercostal muscle per se, that is likely implicated in the oxidative fibre type adaptations. It is also observed that when the rib cage expands as the COPD disease progresses, intercostal muscle shortening is thereby impaired, making patients more reliant on diaphragm ventilation (Laghi & Tobin, 2012). Therefore, a vicious circle of locomotor muscle fatigue, caused by an insufficient oxygen transport because of exaggerated arterial hypoxaemia, and excessive respiratory muscle work (Amann et al., 2010) leading to hyperinflation and rib cage expansion, may arise.

Overall, most of the evidence suggests increased perfusion and oxidative capacity of the respiratory muscles (e.g., the diaphragm) in COPD as opposed to the leg muscles, but whether these changes are adaptive or maladaptive remains equivocal.

## 2 BEYOND MUSCLE DECONDITIONING

Central and peripheral muscle function are affected by COPD, but the question is whether these adaptations to COPD are beneficial for whole‐body function or not. The prevailing hypothesis is that physical inactivity is driving the peripheral muscle loss of function, which also explains the gain of intramuscular metabolic capacity of the diaphragm due to higher inspiratory workload. Over the past two decades, much research has focused on developing countermeasures such as exercise training, nutritional support and more effective smoking cessation strategies. These interventions, which are integral components of pulmonary rehabilitation for COPD, may promote quadriceps muscle adaptation towards a more oxidative profile, but without a definitive mechanism for reducing the symptom burden in COPD patients. We lack data on the plasticity of respiratory muscles in response to interventions like exercise training, and it is unclear what is biologically plausible. Could the diaphragm achieve an even higher oxidative capacity? As COPD progresses, it leads to reduced exercise capacity and physical activity levels, which causes changes in muscles throughout the body. But do these changes occur first or more rapidly in the early stages of COPD, potentially triggering a cycle of inactivity for patients? Perhaps a way to address these unanswered questions is by employing alternative study designs, such as ‘experiments of nature’, which allow for much more extreme or long‐term exposure to physiological interventions than the traditional experiments cited here (Joyner et al., 2023). Such prospective studies in COPD, for example before and after lung transplantation to test the hypothesis that peripheral muscle can regenerate when respiratory deficits are normalized, central versus peripheral muscle adaptations to long‐term exercise training, twin studies or rare genetic abnormalities might expand our understanding of the muscular consequences of COPD. Moreover, the heterogeneity within COPD is further complicated by the frequent presence of comorbidities such as cardiovascular disease including heart failure (Oliveira et al., 2016; Rocha et al., 2019), the use of medications that may affect metabolism, and the presence of low‐grade inflammation. This emphasizes the importance of clearly phenotyping study cohorts and challenges researchers to account for the multiple complexities of the disease.

Much like Aristotle's unanswered question, however, the relationship between COPD and myopathy remains difficult to untangle. Looking at COPD myopathy as a muscle cell disease might be too simplistic, yet intriguing. Investigating the integrative nature of human muscle function, where redundant systems are ready to take over whenever one system is weakened, the future challenge is to determine if selected mechanisms are obligatory for the muscle (mal)adaptations in COPD to develop.

## AUTHOR CONTRIBUTIONS

Jacob Peter Hartmann: First draft, revisions. Hannah G. Caldwell: Figure, revisions. Ulrik W. Iepsen: First draft, revisions, supervision. All authors approved the final version of the manuscript and agree to be accountable for all aspects of the work in ensuring that questions related to the accuracy or integrity of any part of the work are appropriately investigated and resolved. All persons designated as authors qualify for authorship, and all those who qualify for authorship are listed.

## CONFLICT OF INTEREST

None declared.
